# The management of unanticipated difficult airways in children of all age groups in anaesthetic practice - the position paper of an expert panel

**DOI:** 10.1186/s13049-019-0666-7

**Published:** 2019-09-18

**Authors:** Wojciech Walas, Dawid Aleksandrowicz, Maria Kornacka, Tomasz Gaszyński, Ewa Helwich, Marek Migdał, Andrzej Piotrowski, Grażyna Siejka, Tomasz Szczapa, Alicja Bartkowska-Śniatkowska, Zenon P. Halaba

**Affiliations:** 1 Paediatric and Neonatal Intensive Care Unit, University Hospital in Opole, Opole, Poland; 2 Anaesthetic Department, Guy’s and St. Thomas’ NHS Foundation Trust, London, UK; 3 Neonatal and Intensive Care Department, Medical University of Warsaw, Warsaw, Poland; 4 Department of Anaesthesiology and Intensive Therapy, Medical University of Lodz, Lodz, Poland; 5 Clinic of Neonatology and Intensive Neonatal Care, Institute of Mother and Child Care, Warsaw, Poland; 6 Paediatric Intensive Care Unit, Children’s Memorial Health Institute, Warsaw, Poland; 7 Department of Anaesthesia and Intensive Care, Children’s Memorial Health Institute, Warsaw, Poland; 8 Department of Paediatric Anaesthesiology and Intensive Therapy, Wroclaw Medical University, Wroclaw, Poland; 9 Department of Neonatology, Neonatal Biophysical Monitoring and Cardiopulmonary Therapies Research Unit, Poznan University of Medical Sciences, Poznan, Poland; 10 Department of Paediatric Anaesthesiology and Intensive Therapy, Poznan University of Medical Sciences, Poznan, Poland; 11 Institute of Medicine, University of Opole, 48 Oleska Str, 45-052 Opole, Poland

**Keywords:** Difficult intubation, Neonate, Infant, Child and adolescent

## Abstract

Children form a specific group of patients, as there are significant differences between children and adults in both anatomy and physiology. Difficult airway may be unanticipated or anticipated. Difficulties encountered during intubation may cause hypoxia, hypoxic brain injury and, in extreme situations, may result in the patient’s death. There are few paediatric difficult-airway guidelines available in the current literature, and some of these have significant limitations. This position paper, intended for unanticipated difficult airway, was elaborated by the panel of specialists representing the Polish Society of Anaesthesiology and Intensive Care as well as the Polish Neonatal Society. It covers both elective intubation and emergency situations in children in all age groups. An integral part of the paper is an algorithm. The paper describes in detail all stages of the algorithm considering some modification in specific age groups, i.e. neonates.

## Introduction

Despite significant progress in non-invasive techniques, tracheal intubation remains indispensable in airway management for many patients requiring intensive care, and general anaesthesia, and is life-saving in many emergency situations. Difficulties encountered during intubation may cause hypoxia and hypoxic brain injury and, in extreme situations, may result in the patient’s death. The problems arising from difficult intubation (DI) may be anticipated or they may be unexpected. In emergency situations it is usually not possible to assess the risk of difficult airway beforehand and the immediate establishment of a patent artificial airway is a priority. The peculiarity of paediatric population in relation to difficult intubation results from natural anatomical conditions, congenital abnormalities, other pathologies, and the limited availability of airway devices resulting from the small size of paediatric patients. Although problems with intubation in children are not frequent, they are a significant problem due to the potential risks. In comparison with adults, children are characterised by higher oxygen consumption and lower oxygen reserve. This results in worse tolerance of respiratory interruptions and leads to faster desaturation and subsequent bradycardia [1, 2]. Children are a heterogeneous group in terms of both the incidence of difficult intubation and the resulting risks. The risk factors of difficult laryngoscopy in children include: ASA III and IV physical status, Mallampati class III and IV, low Body Mass Index, children intubated for cardiac surgery and maxillofacial surgery [3, 4]. There are many difficult-airway algorithms available in the literature but they have limited applicability to children [5–13]. The presented algorithm is a result of the work of a group of specialists whose activities were endorsed by the Section of Paediatric Anaesthesiology and Intensive Therapy, the Section of Difficult Airways of the Polish Society of Anaesthesiology and Intensive Therapy, and the Polish Neonatal Society. There is insufficient quality data from randomised controlled trials on which to develop evidence-based guidelines and therefore ours are based mostly on a consensus reached by all members of the working group. This algorithm is intended for unanticipated difficult intubation in children for all age groups in various clinical situations while the managing anticipated difficult airway goes beyond this study. It covers both elective and emergency intubation. The guidelines form an integral part of the algorithm.

### Algorithm

**Fig. 1:**
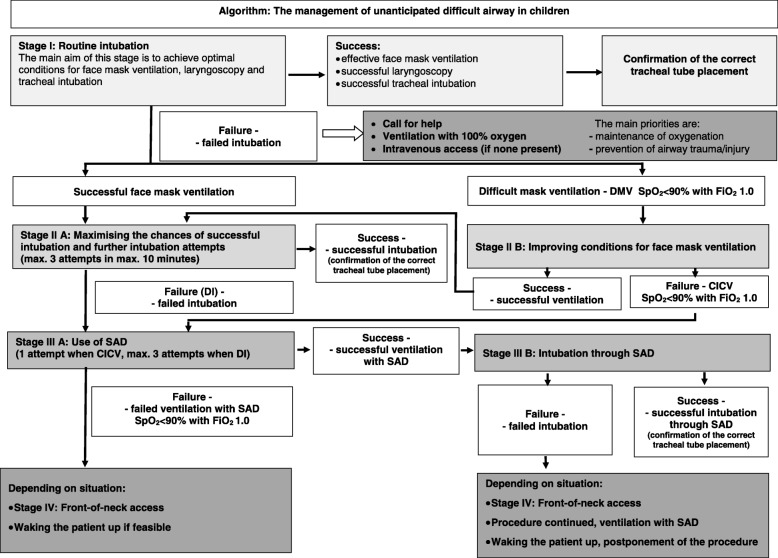
Algorithm: The management of unanticipated difficult airway in children

This algorithm describes the management of unanticipated difficult airway in children. It can be used in all age groups, in both routine induction of anaesthetic for elective cases as well as in emergency situations such as acute respiratory failure. The proposed modifications in the management of different age groups were introduced because of significant differences in both anatomy and physiology of the respiratory system in this population. They may include a rapid move towards Stage IV - front-of-neck access (bypassing other stages) or waking the patient up, if the clinical situation allows this (Fig. 1).

#### Stage I: routine intubation

The principal objective of this stage is to achieve optimal conditions for face mask ventilation, laryngoscopy and tracheal intubation.

It includes:

1. adequate head and neck position
2. choice of an appropriate size and shape of face mask
3. choice of both an adequate laryngoscopy technique and size and shape of the laryngoscope blade
4. choice of an appropriate tracheal tube
5. effective pre-oxygenation
6. removal of upper airway secretions
7. effective anaesthetic and paralysis (in the case of elective intubation and some emergency situations)
8. use of external manoeuvres facilitating intubation
9. use of standard malleable airway stylet or bougie.

Neutral position is recommended in neonates, infants and children up to 2 years of age. Sniffing position is recommended in the remaining age group, i.e. > 2 years old. Both chin lift and jaw trust manoeuvres have been shown to be useful [11, 14, 15]. The choice of an appropriate size and shape of face mask is related to individual patient’s characteristics and should be made on a per-patient basis. The choice of laryngoscopy technique should be individual and depends on personal preference:

1. A straight blade may be used, combined with lifting the epiglottis in order to obtain the laryngeal inlet view. This technique is often used in neonates and infants.
2. In older children, a curved blade is frequently used and placed in the vallecula in order to indirectly lift the epiglottis [15, 16].

No consensus has been reached on whether to use cuffed or uncuffed tracheal tubes in neonates, infants and small children. However, recent studies have shown benefit from, and support the use of, an endotracheal tube containing a high-volume, low-pressure cuff, in all age groups [17–23]. Table 1 shows recommended sizes of tracheal tubes (TTs) for neonates and their depths of insertion [24]. A size 4–4.5 TT is usually used in infants and children up to the age of 2 years and the appropriate size of the TT in older children can be calculated using one of the existing formulae [25–28]. Bag-mask ventilation lasting 3–5 min provides safe apnoea times of 1.5 to 5 min depending on age and the maintenance of a patent upper airway [29–32]. Pre-oxygenation is recommended both in the cases of elective intubation and in the emergency intubation. Pre-oxygenation lasting 60 s through a face mask with oxygen flow of 6 L min^− 1^ is recommended in children aged 5 years and above [16, 33]. Optimal oxygenation with a tight-fitting face mask may be difficult in younger children due to anxiety and lack of cooperation. This problem may be overcome with oxygen being delivered via nasal cannulae. The safe apnoea period can also be extended using oropharyngeal oxygen administration during laryngoscopy [34]. Routine suction of secretions from the upper airways is not recommended, although it should be performed when there are symptoms suggestive of secretion accumulation. In the case of elective intubation and in the emergency intubation, except for situation when a prompt intubation is essential (such as cardiac arrest), intravenous administration of an anticholinergic agent (atropine) is recommended. It decreases mucous secretion in the airways and attenuates vagal reflexes. However, the benefits of such a practice in children are unclear. Secretions from the upper airways should be removed under direct vision. The induction of the anaesthetic as well as the use of neuromuscular blocking agents (NMBAs) should follow the latest anaesthetic guidelines [24, 35]. Adequate anaesthesia is mandatory for successful and atraumatic intubation in the cases of elective intubation and some emergency intubation, except for situations when a prompt intubation is essential (such as cardiac arrest). Opioid administration obtunds sympathetic response to laryngoscopy. The majority of authors have noted the benefits of paralysis in children to improve the conditions for intubation [36–38]. The use of NMBAs is not usually required in neonates and in preterm babies in particular, providing there is adequate anaesthesia [16, 24]. Although the BURP (Backward, Upward, Rightward Pressure) manoeuvre is sometimes recommended for improving laryngoscopy, the use of external manoeuvres should not be uncritically recommended in children [39, 40]. Intubation with a malleable airway stylet might be useful when difficulties in insertion of the TT into the trachea are encountered. The TT should be shaped round the airway stylet and the distal end of the stylet should not extend beyond the distal tip of the TT.

**Table 1:** Recommended tracheal tube size and depth of tracheal tube insertion for oral and nasal intubation in relation to the body weight of a neonate and an infant

Body weight (g)	Internal diameter (mm)	Depth of tracheal tube insertion (oral intubation) (cm)	Depth of tracheal tube insertion (nasal intubation) (cm)
< 750	2.5	5.5–6	6.5–7
750–1000	3	6–7	7–8
1001–2000	3	7–8	9
2001–3000	3.5	8–9	10
3000–3500	3.5	9–10	11
> 3500	4.0	b.w. (kg) + 6	b.w. (kg) + 7

*bw* body weight

At this stage there may be two outcomes:

1. ***Success*** - effective face mask ventilation, laryngoscopy and tracheal tube insertion.

The correct placement of the TT, including the depth of insertion, should be confirmed. As there is no single best method of confirming the tracheal tube placement, the routine approach should initially begin with a direct-vision affirmation during laryngoscopy and intubation. Other methods include verification of symmetrical chest movements during ventilation, symmetrical breath sounds, the condensation of water vapour on the internal wall of the tube during expiration, the lack of auscultation sounds that are typical for oesophageal intubation and the lack of abdominal distension suggestive of air accumulation in the stomach [12]. Auscultation must include three points: bilateral axillae and the epigastrium. Palpation of the suprasternal notch with simultaneous gentle movements of the tracheal tube may also be used to confirm correct TT placement. Capnography should be used to confirm tracheal placement wherever possible [41]. The correct depth of TT insertion may sometimes require radiological confirmation [14]. The tracheal tube placement can also be confirmed with ultrasound [42–46]. A fibre-optic scope may be used for TT placement confirmation in some clinical situations. Some tracheal tubes have a marker that should reach the level of the vocal cords in order to confirm the right depth of TT insertion. In children aged 1 year and above, the correct depth of TT insertion can be calculated using one of the existing formulae [46, 47].

2. ***Failure*** - may result from difficult laryngoscopy, e.g. poor glottic visualisation or (rarely) from difficulty in insertion of the TT into the trachea despite a good laryngoscopic view.

A second experienced (consultant) anaesthetist should be called when a failed intubation is declared. Ventilation should be continued with 100% oxygen (FiO_2_ 1.0). It is of the utmost importance to gain intravenous (IV) access if it has not been done earlier [11–13, 37]. The main priorities for further stages of this algorithm are maintenance of oxygenation and prevention of airway trauma. The ability to ventilate through the face mask will determine further action.

#### Stage II a: Maximising the chances of successful intubation and further intubation attempts (max. Three attempts at laryngoscopy in max. 10 min)

Difficult intubation may not be an immediately life-threatening situation, providing there is effective face mask ventilation. However, as there is always a possibility of difficult mask ventilation, the patient should be ventilated with 100% oxygen [11–13, 37]. The main aim of this stage is to implement all the actions that will maximise the chances of successful intubation (a maximum of three oral intubation attempts allowed). The risk of desaturation may be minimised by insufflation of 100% oxygen during laryngoscopy [34, 48]. We propose a modification of the approach to difficult intubation (DI) in children, limiting the number of attempts at laryngoscopy to three as more appropriate and safer. Furthermore, the duration of all three intubation attempts should be not longer than 10 min [49]. Oral intubation is recommended in this and the following stages of the algorithm. The causes of failure fall into two main groups: difficult laryngoscopy, i.e. visualisation of the laryngeal inlet, or difficulty in tracheal tube insertion into the trachea despite a good laryngoscopic view.

##### Difficult laryngoscopy

The chief aim is to improve the laryngeal inlet view. The relevant actions consist of a sequence of steps:

1. improved head and neck position
2. removal of any secretions from the upper airways
3. effective anaesthesia and paralysis- in the case of elective intubation and some emergency situations (may be deepened, if required)
4. optimal use of external manoeuvres facilitating intubation, e.g. BURP
5. different intubation technique, different size and type of laryngoscope blade
6. use of a different laryngoscope facilitating glottic visualisation, e.g. video laryngoscope
7. use of stylets and introducers
8. use of fibre-optic scope (if readily available)
9. use of ultrasound

Adequate muscle paralysis facilitates laryngoscopy and NMBA administration is considered safe and beneficial in the cases of elective intubation and in the emergency intubation, except for situations when a prompt intubation is essential (such as cardiac arrest) [13, 36, 37]. Optimal NMBAs would be rocuronium or vecuronium, as both of these drugs have a specific reversal agent, i.e. sugammadex (off-label use in children under the age of 2 years) [50]. It is possible to use suxamethonium after contraindication has been excluded. Suxamethonium should be administered with atropine (if it has not been given previously). The glottic visualisation may be improved by external manoeuvres on the larynx, e.g. BURP [39, 40]. A change of the laryngoscope blade, as well as intubation technique, may be useful in neonates and infants. Another option may be the insertion of the laryngoscope blade lateral to the tongue. Video laryngoscopes are becoming more popular and are also recommended in the youngest patients [51–55]. The use of introducers and stylets does not improve laryngoscopic view per se*,* but both of these adjuncts may be useful during tracheal tube insertion into the trachea when the laryngeal inlet is only partially visible. Of note is the fact that in such situations there is a risk of airway injury. This is why such intubation should be performed by an experienced anaesthetist. Both optical stylets and light wands may be used for paediatric intubation, although their usefulness has not been thoroughly assessed [56–59]. The use of a fibre-optic scope is recommended in anticipated difficult airway. They may be used in emergency situations only when readily available and when there is an experienced operator [13, 60–62]. In the current literature there are studies evaluating ultrasound-guided intubation. Such an approach may be considered during difficult intubation when the ultrasound machine/imaging is immediately available and there is a trained operator but there is insufficient evidence to recommend this method routinely [63].

##### Difficult insertion of the tracheal tube despite good laryngoscopic view

This may arise when the tip of the tracheal tube gets stuck in the anterior commissure of the vocal cords. A 90° anticlockwise rotation may solve the problem [14]. Insufficient opening of the laryngeal inlet may also cause problems. It is often due to inadequate anaesthesia and/or paralysis, laryngospasm or anatomical anomalies.

The following actions may facilitate tracheal tube insertion:

1. deepening anaesthesia, effective paralysis- in the case of elective intubation and some emergency situations
2. use of smaller tracheal tubes
3. use of introducers and stylets

Deepening anaesthesia and effective paralysis facilitate intubation when there is insufficient glottic opening or there is laryngospasm in the cases of elective intubation and in the emergency intubation, except for situations when a prompt intubation is essential, such as cardiac arrest (see stage II A, Difficult laryngoscopy) [13, 35, 37]. The use of smaller-size tracheal tubes may be of benefit when there are anatomical obstacles [11]. Prevention of airway injury is of the utmost importance. Intubation with a standard malleable airway stylet is useful when reinforced tubes are used, as the TT may be formed around the stylet. Gum elastic bougie (straight or curved) is useful when there is difficult intubation despite a good laryngoscopic view. Initially the introducer (bougie) is inserted into the trachea and then the tracheal tube is railroaded over it. Care should be undertaken to avoid advancing it too far, especially in smaller children. Other airway adjuncts may also be useful, e.g. optical stylets or light wand, which are available in sizes for older children [56, 57, 64].

At this stage there may be two outcomes:

1. ***Success*** - successful tracheal intubation.
2. ***Failure*** - inability to intubate the patient despite taking all actions/measures aimed at maximising the chances of successful intubation.

The inability to intubate the patient in three consecutive attempts, despite all actions taken to maximise the chances of successful intubation, constitutes difficult intubation.

DI is an indication to move to Stage III.

#### Stage II B: improving conditions for face mask ventilation

Difficult Mask Ventilation (DMV) is indicated by unsatisfactory chest movements and the inability to oxygenate the patient – SpO_2_ < 90% with FiO_2_ 1.0.In contrast with adults, it is rather rare in children [64, 65]. Face mask ventilation attempts should be continued with 100% oxygen, even if there is initial DMV. Oxygen delivery/insufflation during laryngoscopy is recommended. Difficult mask ventilation may occur after induction but before intubation attempts. A rapid attempt to intubate the patient should be made only if the patient is well oxygenated (effective pre-oxygenation, SpO_2_ > 90%). The other causes of DMV include: technical problems (gas/air leak around the face mask) and increased upper or (rarely) lower airway resistance due to functional or anatomical changes. The functional/reversible causes of DMV are: laryngospasm, chest stiffness associated with opioid administration, excessive gastric insufflation with air and bronchospasm [11, 13, 66–68].

The following actions should be taken in order to facilitate face mask ventilation:

1. solving all equipment problems
2. removal of secretions from the upper airways
3. improved head and neck position, application of chin lift and jaw thrust
4. improved seal around the face mask
5. drainage of/emptying the stomach
6. effective anaesthesia and paralysis- in the case of elective intubation and some emergency situations
7. use of oropharyngeal airway or nasopharyngeal airway
8. cautious increase of pressure/volume of inspired air/gas during face mask ventilation
9. administration of a bronchodilator in bronchospasm

Checking the equipment before the start of anaesthesia is an important element of anaesthetic practice [69]. Secretions in the nostrils, oral cavity and pharynx make face mask ventilation difficult and ideally should be removed under direct vision. Changing the shape or size of the face mask may solve the problem. There may be situations in which two hands will be required to achieve an adequate seal around the face mask. In such situations, a two-person technique is recommended [11, 13, 15, 70, 71]. Difficult mask ventilation is often associated with excessive gastric insufflation, which in turn may further compromise ventilation. A nasogastric (NG) tube may be used to empty the stomach and drain the accumulated air/gas [11, 70]. Deepening anaesthesia is often beneficial as it improves ventilation, especially when laryngospasm is present in the cases of elective intubation and in the emergency intubation, except for situations when a prompt intubation is essential (such as cardiac arrest) [11, 13]. The vast majority of authors have concluded that adequate paralysis may facilitate mask ventilation [13, 24, 35, 38, 70]. Oropharyngeal airway facilitates face mask ventilation only when it is correctly inserted and when the right size of OPA is used. The nasopharyngeal airway is used less frequently but is effective in difficult face mask ventilation [72–74]. One of the causes of difficult face mask ventilation is low inspiratory pressure or low-pressure settings of the ‘resuscitator’ (in neonates). A cautious increase in inspiratory pressures/volumes with simultaneous changes in head and neck position may improve face mask ventilation. The use of adrenaline/epinephrine is justified when bronchospasm is the cause of difficult mask ventilation [70].

At this stage there may be two outcomes:

1. ***Success*** - successful face mask ventilation is achieved

In this situation, there is no immediate threat to life. This warrants the move to Stage IIA.

2. ***Failure*** - no improvement of face mask ventilation (no chest movements, SpO_2_ < 90% with FiO_2_ 1.0) despite taking all the above-mentioned actions/measures

In this case, the inability to intubate and ventilate the patient should be declared (CICV - Cannot Intubate, Cannot Ventilate). It is a life-threatening situation and warrants a prompt move to Stage III A. Attempts to ventilate the patient with 100% oxygen should continue.

#### Stage III a: rescue use of Supraglottic Airway device

maximum three attempts allowed.

Failure to intubate when face mask ventilation is possible does not constitute a life-threatening situation. In contrast, failure to intubate and the inability to ventilate the patient through a face mask is an immediate threat to life (CICV). In this case, prompt actions should be initiated and attempts to ventilate the patient with 100% oxygen should be continued. The most popular Supraglottic Airway Devices (SAD) are Laryngeal Mask Airways (LMA) and their various modifications, e.g. ILMA - Intubating Laryngeal Mask Airway. The most useful SADs are those which can be used not only for ventilation but also for intubation when the routine intubation is difficult. In the neonatal group, SADs are recommended in patients with a corrected gestational age of 34 weeks and above, and with body weight over 2000 g [15, 75–77]. Table 2 shows recommended sizes of LMAs for children, but the recommended sizing varies between different devices [78]. The Combitube device may be used in children who are taller than 120 cm, i.e. school-aged children. These devices enable mechanical ventilation and some of them, e.g. the iLTS-D, are designed to facilitate fibre-optic-assisted tracheal intubation.

**Table 2:** Recommended LMA sizing

Patient size	LMA size
Neonate, infant < 5 kg	1
Infant 5–10 kg	1.5
Infant, child 10–20 kg	2
Child 20–30 kg	2.5
Child 30–50 kg	3
Child /Adult 50–70 kg	4.0
Child / Adult 70–100 kg	5

There may be two outcomes at this stage:

1. ***Success*** - successful ventilation with an SAD

This warrants the move to Stage III B.

2. ***Failure*** - inability to ventilate through an SAD

The inability to ventilate through an SAD, together with the inability to intubate and ventilate the patient (CICV), indicates a life-threatening situation and warrants a prompt move to Stage IV. Attempts to ventilate the patient with 100% oxygen should continue. Inability to ventilate through an SAD is not an immediate threat to life when face mask ventilation is possible. In this situation, face mask ventilation should be continued, and further actions depend on the clinical circumstances:

- A prompt move to Stage IV in all situations requiring artificial airway for patient management,
- Waking the patient up, if feasible.

In some cases, an attempt to wake the patients up may pose the risk for them and in that situation a prompt move to Stage IV is required.

#### Stage III B: intubation trough an SAD

The absolute need for tracheal intubation should be reviewed. In some situations, surgery should be abandoned, and the child woken up; in others the procedure may be carried out using an SAD or intubation through an SAD can be attempted.

The classic LMA is mainly used for mechanical ventilation, although it is possible to intubate through it. The use of a fibre-optic scope is recommended for both intubation through the SAD and the confirmation of the tracheal tube placement [79–85]. Blind intubation through an SAD should be avoided if at all possible [86, 87].

There may be two outcomes at this stage:

1. ***Success*** - successful intubation through SAD.
2. ***Failure*** - failed intubation through SAD.

In this situation, ventilation through SAD should be continued and further actions depend on the clinical circumstances:

- A prompt move to Stage IV in all situations requiring artificial airway for patient management,
- There are three options available when there is a failed intubation during elective intubation:
  1. Move to Stage IV for all urgent/emergency procedures that cannot be done under general anaesthetic with ventilation through SAD.
  2. Continuing surgery under general anaesthetic with ventilation through SAD - for shorter procedures and with the patient in the supine position.
  3. Waking the patient up and postponement of the surgical procedure if feasible.

#### Stage IV: front-of-neck access

The anterior neck approach comprises the following methods:

1. needle cricothyroidotomy
2. surgical cricothyroidotomy
3. retrograde intubation
4. tracheotomy

Front-of-neck access is rarely used in children. In older children, dedicated sets are used and, in an emergency, a substitute instrument, such as intravenous cannulae, may be utilised [88–90]. In life-threatening situations when bag-mask ventilation and ventilation through Laryngeal Mask Airway is not possible, an option to achieve the quickest-possible patient oxygenation should be chosen, depending on the available equipment and physician’s experience. It has to be taken into account, however, that cricoid puncture and cricothyroidotomy is associated with high risk in children - the smaller the patient, the higher the risk [11, 91–93]. Tracheotomy is relatively safe when performed by an experienced surgeon (general or ENT surgeon). It is, however, more time-consuming [11, 13]. Needle cricothyroidotomy is the fastest front-of-neck access. If this method is chosen, high respiratory resistance should be anticipated, especially during expiration, which should be given enough time [11, 94–96]. Jet ventilation (high pressure, low volume) by means of special devices is proposed [11, 97]. Its use is possible when at least a minimal leak upwards from cannula insertion is present. Barotrauma with this technique is a real life-threatening problem and the technique is contraindicated if there is no expiration route. Surgical cricothyroidotomy (Melker kit or scalpel bougie technique) requires more time to perform than needle cricothyroidotomy but enables efficient ventilation of the patient [89]. Retrograde intubation is rarely performed and requires piercing the cricothyroid membrane (alternatively cricotracheal or between the tracheal cartilages) cephalad with a needle, threading a guidewire upwards toward the patient’s mouth and railroading the endotracheal tube over the guidewire into the trachea. Retrograde intubation can be combined with a fibre-optic scope and with ventilation through an LMA to avoid breaks in the patient’s ventilation [98–102]. Tracheotomy can be performed at any age but there are very limited indications for it in neonates [11, 13, 103]. It is an effective way to achieve proper ventilation of a patient and, in experienced hands, is the safest approach through the anterior neck [11, 13].This procedure is recommended as elective but it is too time-consuming for emergency situations. Most techniques require a fibre-optic control to confirm the proper position of the tracheostomy tube. Special percutaneous tracheotomy sets are available in sizes for adults; therefore, this method has a limited application in children [89]. In the immediate life-threatening situation with CICV/CICO (Cannot Intubate, Cannot Oxygenate) and lack of appropriate equipment, it is advisable to pierce the cricothyroid membrane (or cricotracheal ligament or between tracheal cartilages) with any available type of cannula. Once this is performed, oxygen should immediately be administered through it.

##### Difficult-airway kit

Table 3 contains the proposed difficult-airway kit. It corresponds to all the stages of the algorithm.

**Table 3:** Difficult airway kit^a^

Stage II A: Maximising the chances of successful intubation. Stage II B: Improving conditions for face mask ventilation.	
1	Specialist/alternative laryngoscopes e.g. McCoy, optical laryngoscope, and/or video laryngoscope and/or optical stylet.
2	Full set of malleable airway stylets and introducers e.g. gum elastic bougie
3	Full set of oropharyngeal airways (in various sizes), additionally recommended full set of nasopharyngeal airways (in various sizes).
4	Tables containing recommended sizes of tracheal tubes and recommended depth of their insertion (age-related).
5	Drugs: rocuronium, suxamethonium, atropine, sugammadex.
Stage III A: Use of SAD Stage III B: Intubation through SAD	
6	Full set of supraglottic airways devices of in various sizes, preferred double-lumen laryngeal mask airways (LMA, ILMA) with a gastric channel.
7	Table containing recommended sizes of LMA.
Stage IV: Front-of-neck access.	
8	Original cricothyroidotomy kit^b^ e.g. Quicktrach, Mini-Trach in various sizes, and/or 6G intravenous cannula for alternative needle cricothyroidotomy.
9	Original retrograde intubation set e.g. Cook® Retrograde Intubation Set in various sizes and/or Tuohy needle and cannulation set (Seldinger technique) for alternative retrograde intubation.
10	Full set of tracheostomy tubes in various sizes.
11	Additional equipment: scalpel, sterile gauze/swab, skin disinfectant, sterile gloves in various sizes.
Additionally recommended.	
12	Manujet III device.
13	Fibre-optic scope with complete kit of different sizes (essential for the management of anticipated difficult airway).

^a^Not all types/models of devices are essential for this kit. A single device from each group is sufficient

^b^“Original kit” here refers to a set of equipment manufactured by medical companies (OEMs), readily available on the market

## Data Availability

Not applicable.

## Abbreviations

BURP: Backward, Upward, Rightward Pressure
CICO: Cannot Intubate, Cannot Oxygenate
CICV: Cannot intubate, cannot ventilate
DA: Difficult airway
DI: Difficult intubation
DMV: Difficult Mask Ventilation
ILMA: Intubating Laryngeal Mask Airway
IV: Intravenous
LMA: Laryngeal Mask Airway
NMBAs: Neuromuscular blocking agents
OPA: Oropharyngeal airway
SAD: Supraglottic airway device
TT: Tracheal tube

## References

[1] Sands SA, Edwards BA, Kelly VJ, Davidson MR, Wilkinson MH, Berger PJ (2010). A model investigation of the impact of ventilation-perfusion mismatch on oxygenation during apnea in preterm infants. J Theor Biol.

[2] Hardman JG, Wills JS (2006). The development of hypoxaemia during apnoea in children: a computational modelling investigation. Br J Anaesth.

[3] Heinrich S, Birkholz T, Ihmsen H (2012). Incidence and predictors of difficult laryngoscopy in 11,219 pediatric anesthesia procedures. Paediatr Anaesth.

[4] Heinrich S, Birkholz T, Ihmsen H (2013). Incidence and predictors of poor laryngoscopic view in children undergoing pediatric cardiac surgery. J Cardiothorac Vasc Anesth.

[5] Frerk C, Mitchell VS, McNarry AF (2015). I difficult Airway society intubation guidelines working group: difficult Airway society 2015 guidelines for management of unanticipated difficult intubation in adults. Br J Anaesth.

[6] Myatra SN, Shah A, Kundra P (2016). All India difficult Airway association 2016 guidelines for the management of unanticipated difficult tracheal intubation in adults. Indian J Anaesth.

[7] Apfelbaum JL, Hagberg CA, Caplan RA (2013). American Society of Anesthesiologists Task Force on Management of the Difficult Airway: practice guidelines for management of the difficult airway: an updated report by the American Society of Anesthesiologists Task Force on management of the difficult Airway. Anesthesiology.

[8] Law JA, Broemling N, Cooper RM (2013). The difficult airway with recommendations for management--part 1--difficult tracheal intubation encountered in an unconscious/induced patient. Can J Anaesth.

[9] Law JA, Broemling N, Cooper RM (2013). The difficult airway with recommendations for management - part 2 - the anticipated difficult airway. Can J Anaesth.

[10] Chrimes N (2016). The vortex: a universal ‘high-acuity implementation tool’ for emergency airway management. Br J Anaesth.

[11] Black AE, Flynn PE, Smith HL, Thomas ML, Wilkinson KA, Association of Pediatric Anaesthetists of Great Britain and Ireland (2015). Development of a guideline for the management of the unanticipated difficult airway in pediatric practice. Paediatr Anaesth.

[12] Pawar DK, Doctor JR, Raveendra US (2016). All India difficult Airway association 2016 guidelines for the management of unanticipated difficult tracheal intubation in Paediatrics. Indian J Anaesth.

[13] Weiss M, Engelhardt T (2010). Proposal for the management of the unexpected difficult pediatric airway. Paediatr Anaesth.

[14] Marraro G, Bissonnette B (2011). Airway management. Pediatric anesthesia.

[15] Airway management and ventilation. In: Wyllie J ed. Newborn Life Support. Course Manual. ERC Guidelines 2015 Edition. European Resuscitation Council vzw, Emile Vanderveldelaan 35, 2845 Niel, Belgium. ISBN 9789079157846

[16] Lönnqvist P-A, Bissonnette B (2011). Management of the Neonate: anesthetic considerations. Pediatric anesthesia.

[17] Litman RS, Maxwell LG (2013). Cuffed versus uncuffed endotracheal tubes in pediatric anesthesia: the debate should finally end. Anesthesiology.

[18] Shi F, Xiao Y, Xiong W, Zhou Q, Huang X (2016). Cuffed versus uncuffed endotracheal tubes in children: a meta-analysis. J Anesth.

[19] De Orange FA, Andrade RG, Lemos A, Borges PS, Figueiroa JN, Kovatsis PG (2017). Cuffed versus uncuffed endotracheal tubes for general anaesthesia in children aged eight years and under. Cochrane Database Syst Rev.

[20] Thomas RE, Rao SC, Minutillo C, Hullett B, Bulsara MK (2018). Cuffed endotracheal tubes in infants less than 3 kg: a retrospective cohort study. Paediatr Anaesth.

[21] Chand R, Roy Chowdhury S, Rupert E, Mandal CK, Narayan P (2018). Benefits of using high-volume–low-pressure tracheal tube in children undergoing congenital cardiac surgery: evidence from a prospective randomized study. Semin Cardiothorac Vasc Anesth.

[22] Thomas R, Rao S, Minutillo C (2016). Cuffed endotracheal tubes for neonates and young infants: a comprehensive review. Arch Dis Child Fetal Neonatal Ed.

[23] Weiss M, Dullenkopf A, Fisher IE, Keller C, Gerber AC (2009). Prospective randomized controlled multi-Centre trial of cuffed or uncuffed endotracheal tubes in small children. Br J Anaesth.

[24] Manowska M, Bartkowska-Śniatkowska A, Zielińska M (2013). The consensus statement of the Paediatric section of the polish Society of Anaesthesiology and Intensive Therapy on general anaesthesia in children under 3 years of age. Anaesthesiol Intensive Ther.

[25] Veyckemans F, Bissonnette B (2011). Anesthesia equipment. Pediatric anesthesia.

[26] Moloney G, Bissonnette B (2011). Which endotracheal tube in neonates, infants, and small children?. Pediatric anesthesia.

[27] Khine HH, Corddry DH, Kettrick RG (1997). Comparison of cuffed and uncuffed endotracheal tubes in young children during general anesthesia. Anesthesiology.

[28] Deakers TW, Reynolds G, Stretton M, Newth CJ (1994). Cuffed endotracheal tubes in pediatric intensive care. J Pediatr.

[29] Patel R, Lenczyk M, Hannallah RS, McGill WA (1994). Age and the onset of desaturation in apnoeic children. Can J Anaesth.

[30] Hardman JG, Willis JS (2006). The development of hypoxaemia during apnoea in children: a computational modeling investigation. Br J Anaesth.

[31] Hardman JG, Wills JS, Aitkenhead AR (2000). Factors determining the onset and course of hypoxaemia during apnoea: an investigation using physiological modeling. Anesth Analg.

[32] Farmery AD, Roe PG (1996). A model to describe the rate of oxyhaemoglobin desaturation during apnoea. Br J Anaesth.

[33] Morrison JE, Collier E, Friesen RH, Logan L (1998). Preoxygenation before laryngoscopy in children: how long is enough?. Paediatr Anaesth.

[34] Steiner JW, Sessler DI, Makarova N (2016). Use of deep laryngeal oxygen insufflation during laryngoscopy in children: a randomized clinical trial. Br J Anaesth.

[35] Karsli C, Issac LA, Bissonnette B (2011). Induction of anesthesia. Pediatric anesthesia.

[36] Ikeda A, Isono S, Sato Y (2012). Effects of muscle relaxants on mask ventilation in anesthetized persons with normal upper airway anatomy. Anesthesiology.

[37] Weiss M, Engelhardt T (2012). Cannot ventilate-paralyze!. Paediatr Anaesth.

[38] Walker RW, Ellwood J (2009). The management of difficult intubation in children. Paediatr Anaesth.

[39] Lerman J, Creighton RE (2006). Two hands, three sites: show me the vocal cords. Paediatr Anaesth.

[40] Kojima T, Laverriere EK, Owen EB (2018). Clinical impact of external laryngeal manipulation during laryngoscopy on tracheal intubation success in critically ill children. Pediatr Crit Care Med.

[41] Bhende MS, Thompson AE, Cook DR, Saville AL (1992). Validity of a disposable end-tidal CO2 detector in verifying endotracheal tube placement in infants and children. Ann Emerg Med.

[42] Chun R, Kirkpatrick AW, Sirois M (2004). Where's the tube? Evaluation of hand-held ultrasound in confirming endotracheal tube placement. Prehosp Disaster Med.

[43] Dennington D, Vali P, Finer NN, Kim JH (2012). Ultrasound confirmation of endotracheal tube position in neonates. Neonatology.

[44] Sethi A, Nimbalkar A, Patel D, Kungwani A, Nimbalkar S (2014). Point of care ultrasonography for position of tip of endotracheal tube in neonates. Indian Pediatr.

[45] Jaeel P, Sheth M, Nguyen J (2017). Ultrasonography for endotracheal tube position in infants and children. Eur J Pediatr.

[46] Yates AP, Harries AJ, Hatch DJ (1987). Estimation of nasotracheal tube length in infants and children. Br J Anaesth.

[47] Lau N, Playfor SD, Rashid A, Dhanarass M (2006). New formulae for predicting tracheal tube length. Paediatr Anaesth.

[48] Windpassinger M, Plattner O, Gemeiner J (2016). Pharyngeal oxygen insufflation during AirTraq laryngoscopy slows arterial desaturation in infants and small children. Anesth Analg.

[49] Lee JH, Turner DA, Kamat P (2016). The number of tracheal intubation attempts matter! A prospective multi-institutional pediatric observational study. BMC Pediatr.

[50] Wołoszczuk-Gębicka B, Zawadzka-Głos L, Lenarczyk J, Sitkowska BD, Rzewnicka I (2014). Two cases of the “cannot ventilate, cannot intubate” scenario in children in view of recent recommendations. Anaesthesiol Intensive Ther.

[51] Owada G, Mihara T, Inagawa G, Asakura A, Goto T, Ka K (2017). A comparison of the Airtraq®, McGrath®, and Macintosh laryngoscopes for difficult paediatric intubation: a manikin study. PLoS One.

[52] Dwivedi D, Bhatnagar V, Tandon U, Jinjil K (2016). Pediatric difficult intubation in a rare genetic disorder made easy with Airtraq laryngoscope. Anesth Essays Res.

[53] Redel A, Karademir F, Schlitterlau A (2009). Validation of the GlideScope video laryngoscope in pediatric patients. Paediatr Anaesth.

[54] Macnair D, Baraclough D, Wilson G, Bloch M, Engelhardt T (2009). Pediatric airway management: comparing the Berci-Kaplan video laryngoscope with direct laryngoscopy. Paediatr Anaesth.

[55] Vlatten A, Aucoin S, Litz S, Macmanus B, Soder C (2009). A comparison of the STORZ video laryngoscope and standard direct laryngoscopy for intubation in the pediatric airway – a randomized clinical trial. Paediatr Anaesth.

[56] Jansen AH, Johnston G (2008). The Shikani optical stylet: a useful adjunct to airway management in a neonate with popliteal pterygium syndrome. Paediatr Anaesth.

[57] Shukry M, Hanson RD, Koveleskie JR, Ramadhyani U (2005). Management of the difficult pediatric airway with Shikani optical stylet. Paediatr Anaesth.

[58] Xue FS, Luo MP, Liao X, Zhang YM (2009). Trachlight guided airway topical anesthesia in children with difficult airways. Paediatr Anaesth.

[59] Xue FS, Liu JH, Zhang YM, Liao X (2009). The lightwand-guided digital intubation in newborns and infants with difficult airways. Paediatr Anaesth.

[60] Vlatten A, Aucoin S, Litz S, MacManus B, Soder C (2010). A comparison of bonfils fiberscope-assisted laryngoscopy and standard direct laryngoscopy in simulated difficult pediatric intubation: a manikin study. Paediatr Anaesth.

[61] Fiadjoe JE, Hirschfeld M, Wu S (2015). A randomized multi-institutional crossover comparison of the GlideScope® cobalt video laryngoscope to the flexible fiberoptic bronchoscope in a Pierre Robin manikin. Paediatr Anaesth.

[62] Liu GP, Li RP, Xue FS (2015). Comparing intubation performance of Bonfils fiberscope and fiberoptic bronchoscope in difficult pediatric airways. Paediatr Anaesth.

[63] Fiadjoe JE, Stricker P, Gurnaney H (2012). Ultrasound-guided tracheal intubation: a novel intubation technique. Anesthesiology.

[64] Sunder RA, Haile DT, Farrell PT, Sharma A (2012). Pediatric airway management: current practices and future directions. Paediatr Anaesth.

[65] Tong DS, Beus J, Liman RS (2007). The Childrens’s Hospital of Philadelphia Difficult Intubation Registry. Anesthesiology.

[66] Cook TM, Woodall N, Frerk C (2011). Fourth National Audit Project. Major complications of airway management in the UK: results of the fourth National Audit Project of the Royal College of Anaesthetists and the difficult Airway society. Part 1: anaesthesia. Br J Anaesth.

[67] von Ungern-Sternberg BS, Boda K, Chambers NA (2010). Risk assessment for respiratory complications in paediatric anaesthesia: a prospective cohort study. Lancet.

[68] Mamie C, Habre W, Delhumeau C, Argiroffo CB, Morabia A (2004). Incidence and risk factors of perioperative respiratory adverse events in children undergoing elective surgery. Paediatr Anaesth.

[69] Kusumaphanyo C, Charuluxananan S, Sriramatr D, Pulnitiporn A, Sriraj W (2009). The Thai anesthesia incident monitoring study (Thai AIMS) of anesthetic equipment failure/malfunction: an analysis of 1996 incident reports. J Med Assoc Thail.

[70] Engelhardt T, Weiss M (2012). A child with a difficult airway: what do I do next?. Curr Opin Anaesthesiol.

[71] Tracy MB, Klimek J, Coughtrey H (2011). Mask leak in one-person mask ventilation compared to two-person in newborn infant manikin study. Arch Dis Child Fetal Neonatal Ed.

[72] Parhizkar N, Saltzman B, Grote K (2011). Nasopharyngeal airway for management of airway obstruction in infants with micrognathia. Cleft Palate Craniofac J.

[73] Roberts K, Whalley H, Bleetman A (2005). The nasopharyngeal airway: dispelling myths and establishing the facts. Emerg Med J.

[74] Abel F, Bajaj Y, Wyatt M, Wallis C (2012). The successful use of the nasopharyngeal airway in Pierre Robin sequence: an 11-year experience. Arch Dis Child.

[75] Bradley AE, White MC, Engelhardt T, Bayley G, Beringer RM (2013). Current UK practice of pediatric supraglottic airway devices - a survey of members of the Association of Paediatric Anaesthetists of Great Britain and Ireland. Paediatr Anaesth.

[76] Schmölzer GM, Agarwal M, Kamlin CO, Davis PG (2013). Supraglottic airway devices during neonatal resuscitation: an historical perspective, systematic review and meta-analysis of available clinical trials. Resuscitation.

[77] Galderisi A, De Bernardo G, Lorenzon E, Trevisanuto D (2015). i-gel: a new supraglottic device for effective resuscitation of a very low birthweight infant with Cornelia de Lange syndrome. BMJ Case Rep.

[78] Classic Laryngeal Mask Airway. Product information on manufacturer’s website. http://www.teleflex.com/emea/documentLibrary/documents/940691-000001_31817-LMA-TF-Classic-A4_1403_PDF.pdf [Accessed 19 Apr 2017].

[79] Reynolds PI, O'Kelly SW (1993). Fiberoptic intubation and the laryngeal mask airway. Anesthesiology.

[80] Benumof JL (2001). A new technique of fiberoptic intubation through a standard LMA. Anesthesiology.

[81] Weiss M, Schwarz U, Dillier C, Fischer J, Gerber AC (2001). Use of the intubating laryngeal mask in children: an evaluation using video-endoscopic monitoring. Eur J Anaesthesiol.

[82] Jöhr M, Berger TM (2004). Fiberoptic intubation through the laryngeal mask airway (LMA) as a standardized procedure. Paediatr Anaesth.

[83] Weiss M, Mauch J, Becke K, Schmidt J, Jöhr M (2009). Fibre optic-assisted endotracheal intubation through the laryngeal mask in children. Anaesthesist.

[84] Walker RW (2000). The laryngeal mask airway in the difficult paediatric airway: an assessment of positioning and use in fibreoptic intubation. Paediatr Anaesth.

[85] Zhi J, Deng XM, Yang D (2016). Comparison of the Ambu Aura-i with the air-Q intubating laryngeal Airway as a conduit for Fiberoptic-guided tracheal intubation in children with ear deformity. Zhongguo Yi Xue Ke Xue Yuan Xue Bao.

[86] Thomas PB, Parry MG (2001). The difficult paediatric airway: a new method of intubation using the laryngeal mask airway, Cook airway exchange catheter and tracheal intubation fibrescope. Paediatr Anaesth.

[87] Selim M, Mowafi H, Al-Ghamdi A, Adu-Gyamfi Y (1999). Intubation via LMA in pediatric patients with difficult airways. Can J Anaesth.

[88] Newgard CD, Koprowicz K, Wang H (2009). Variation in the type, rate, and selection of patients for out-of-hospital airway procedures among injured children and adults. Acad Emerg Med.

[89] Coté CJ, Hartnick CJ (2009). Pediatric transtracheal and cricothyrotomy airway devices for emergency use: which are appropriate for infants and children?. Paediatr Anaesth.

[90] Advanced Paediatric Life Support: A Practical Approach to Emergencies, 6th Edition. Chichester: Wiley. 2016. ISBN: 9781118947647.

[91] Blanas N, Fisher JA (1999). A “last ditch” airway, revisited. Can J Anaesth.

[92] Navsa N, Tossel G, Boon JM (2005). Dimensions of the neonatal cricothyroid membrane - how feasible is a surgical cricothyroidotomy?. Paediatr Anaesth.

[93] Corbett HJ, Mann KS, Mitra I, Jesudason EC, Losty PD, Clarke RW (2007). Tracheostomy – a 10-year experience from a UK pediatric surgical center. J Pediatr Surg.

[94] Chong CF, Wang TL, Chang H (2003). Percutaneous transtracheal ventilation without a jet ventilator. Am J Emerg Med.

[95] Zornow MH, Thomas TC, Scheller MS (1989). The efficacy of three different methods of transtracheal ventilation. Can J Anaesth.

[96] Steward DJ (1987). Percutaneous transtracheal ventilation for laser endoscopic procedures in infants and small children. Can J Anaesth.

[97] Li S, Liu Y, Tan F, Chen J, Chen L (2010). Efficacy of manual jet ventilation using Manujet III for bronchoscopic airway foreign body removal in children. Int J Pediatr Otorhinolaryngol.

[98] Sanguanwit P, Trainarongsakul T, Kaewsawang N, Sawanyawisuth K, Sitthichanbuncha Y (2016). Is retrograde intubation more successful than direct laryngoscopic technique in difficult endotracheal intubation ?. Am J Emerg Med.

[99] Ciftci T, Erbatur S (2016). Retrograde intubation via laryngeal mask airway in a paediatric patient with fallot-type ventricular septal defect and cleft palate deformity. Middle East J Anaesthesiol.

[100] Miner JR, Rubin J, Clark J, Reardon RF (2015). Retrograde intubation with an Extraglottic device in place. J Emerg Med.

[101] He M (2014). Emergent retrograde tracheal intubation in a 3-year-old with stevens-johnsons syndrome. A A Case Rep.

[102] Cui XL, Wang SY, Xue FS (2014). Fiberoptic and retrograde intubation in difficult pediatric airway: useful suggestions. J Neurosurg Anesthesiol.

[103] McLaughlin J, Iserson KV (1986). Emergency pediatric tracheostomy: a usable technique and model for instruction. Ann Emerg Med.

